# Proximal femoral replacement with locking plate for massive bone loss: a case report

**DOI:** 10.1051/sicotj/2025024

**Published:** 2025-05-12

**Authors:** Hironori Ochi, Tomonori Baba, Masahiko Nozawa, Suguru Kato, Kyoko Sasaki, Yuko Sakamoto, Sung-Gon Kim, Muneaki Ishijima

**Affiliations:** 1 Department of Orthopaedic Surgery, Juntendo University Nerima Hospital 3-1-10, Takanodai Nerima-ku Tokyo 177-8521 Japan; 2 Department of Orthopaedic Surgery, Juntendo University School of Medicine 2-1-1 Hongo Bunkyo-ku Tokyo 113-0033 Japan

**Keywords:** Massive proximal femoral bone loss, Femoral reconstruction, Proximal femoral replacement, Locking plate, Stem loosening

## Abstract

Complications on the femoral side after performing proximal femoral replacement (PFR), such as stem loosening and periprosthetic fractures, are the major reasons for reoperation. Femoral reconstruction was performed using PFR with a locking plate to minimize the risk of complications. We present the case of an 85-year-old woman with stem loosening and massive proximal femoral bone loss (Paprosky type IV) 10 years after stem revision in bipolar hemiarthroplasty. Femoral reconstruction was performed using the following surgical techniques. After removing the previous implant, a PFR was inserted into the host bone of the distal femur and fixed at the junction with cement. In addition, a locking plate was used for bridging. Full weight-bearing rehabilitation was started the day after surgery. At the 5-year follow-up, the patient could walk steadily without complications. A postoperative radiograph of the femur showed no signs of a radiolucent line, implant-related issues, or bone resorption. This reconstructive technique may reduce the high torsional and compressive stresses on bone cement prostheses, which can cause complications on the femoral side. Even in the case of poor femoral host bone quality, this reconstruction method can achieve robust femoral reconstruction. Femorl reconstruction using PFR with a locking plate is a particularly beneficial reconstruction method for older patients with massive proximal femoral bone loss.

## Introduction

Massive proximal femoral bone loss is complicated in both neoplastic and non-neoplastic conditions, such as failed arthroplasty, infection, complex trauma, periprosthetic femoral fracture (PFF), or multiple attempts at osteosynthesis [1]. Proximal femoral replacement (PFR), allograft prosthetic composite (APC), and long revision stems are the primary treatment options for massive proximal femoral bone loss [2]. PFR has two advantages over the other methods: the surgical approach is rather simple, and it allows for early weight bearing because bone healing is unnecessary [2]. However, the use of PFR has a high rate of complications, including dislocation, stem loosening, prosthesis failure, PFF, and infection [3]. While APC has the remarkable benefits of restoring and improving bone stock and reattaching soft tissues to prevent instability, it has a higher complication rate than the other methods [2]. Even though revision stems have a lower complication rate, they should only be used for specific bone conditions [2].

Owing to the positive outcomes observed in neoplastic conditions utilizing PFR, the indication of its use was broadened for non-neoplastic patients with massive femoral bone loss encountered during complex revision total hip arthroplasty (THA) [4]. In this case, the patient was an older adult, and we considered PFR, a favorable option as it allows early weight-bearing. However, complications on the femoral side when using PFR, such as stem loosening and PFF, remain major reasons for reoperation [2, 4]. Especially under conditions where the quality of the remaining distal femur is poor, the use of PFR alone is considered to increase the risk of loosening and PFF. We hypothesized that adding a locking plate to bridge the PFR and distal femoral host bone might reduce torsional and compressive stresses at the bone-cement-prosthesis junction, thereby helping to mitigate these complications. To reduce the risk of these complications, we performed a femoral reconstruction using PFR with a locking plate for massive proximal femoral bone loss.

## Case

This study was approved by the Medical Research Ethics Committee of Juntendo University Nerima Hospital (approval number S24-08) and conducted according to the Declaration of Helsinki.

**Figure 1:**
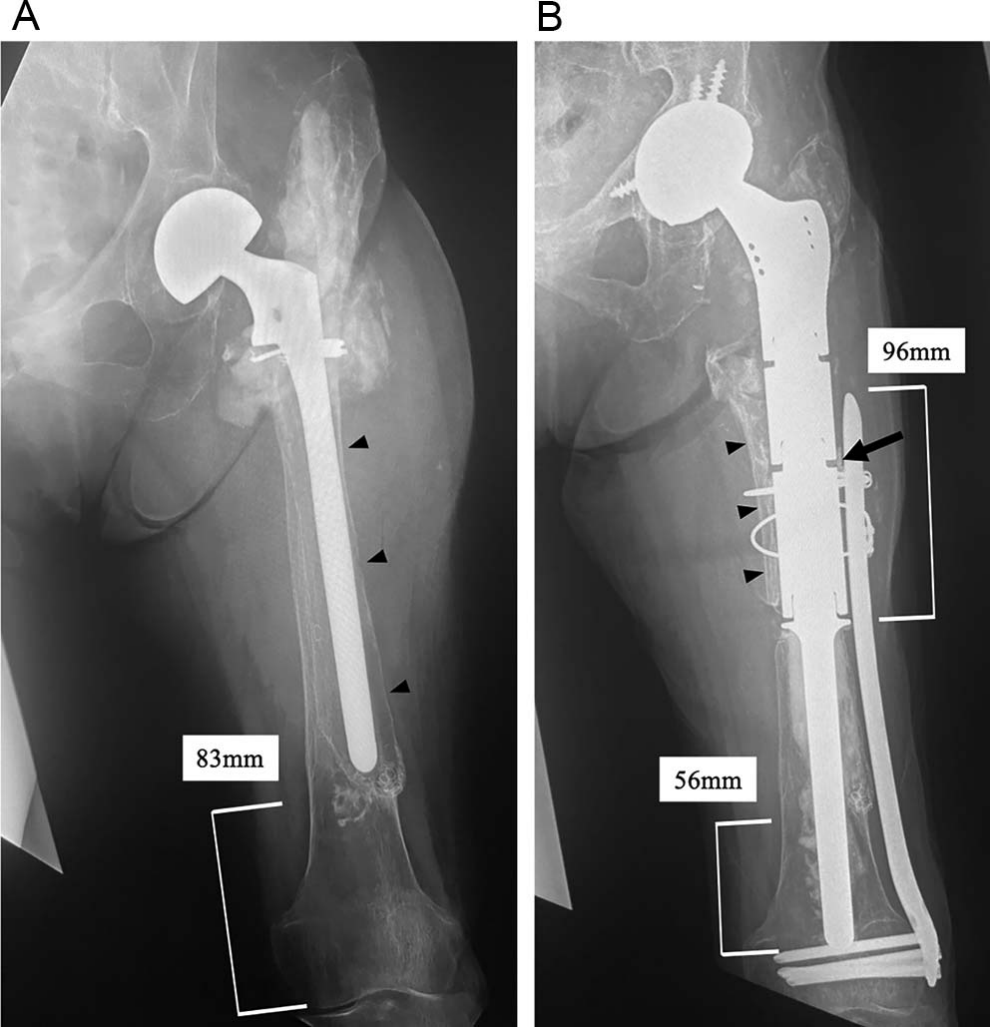
(A) A preoperative radiograph of the femur demonstrating stem loosening and massive bone loss from the proximal to the distal end of the diaphyseal femur (Paprosky type IV) with acetabular osteolysis on the left side. The radiolucent line surrounding the stem and thin cortical bone of the femur are observed (*black arrowheads*). Additionally, severe stem migration as a consequence of the loosening is identified. The intact distal femoral bone remains only approximately 83 mm from the distal end of the femur. In addition, its bone quality is osteoporotic. (B) A postoperative radiograph of the femur 5 years after surgery demonstrates no signs of a radiolucent line, implant-related issues, or bone resorption. The PFR stem is inserted 56 mm into the intact distal femur (approximately 45% of the PFR stem length). A locking plate is placed on the femur, overlapping the PFR 96 mm proximally from the bone-cement-prosthesis junction. The black arrowheads indicate the longitudinally opened bone fragment of the proximal diaphyseal femur. The black arrow indicates the free cortical bone fragment between the PFR and locking plate.

**Figure 2:**
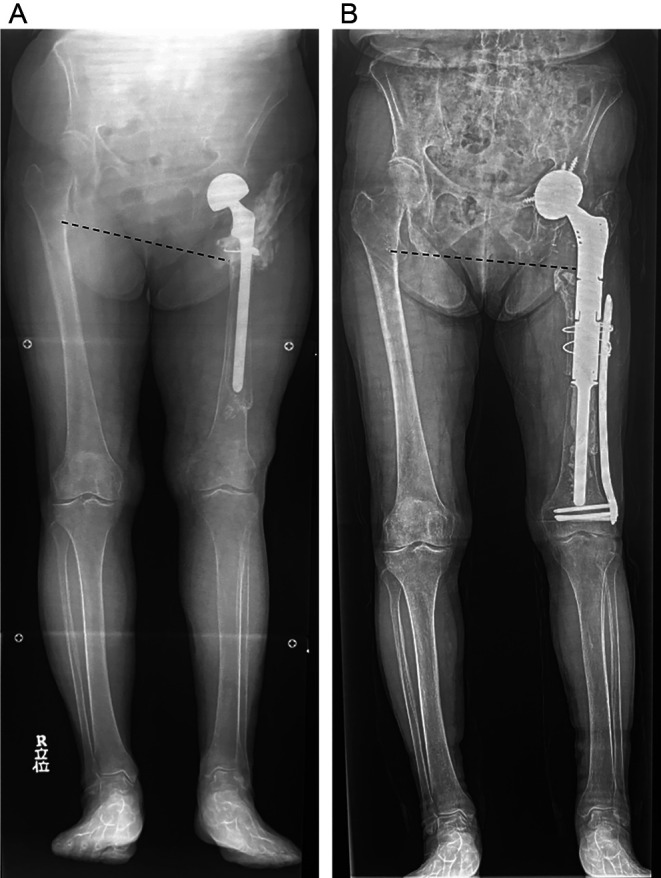
(A) A preoperative radiograph of the full length of the lower limbs demonstrates a severe leg-length discrepancy due to shortening of the femur. Severe pelvic obliquity is observed (*dotted line*). (B) A postoperative radiograph of the full length of the lower limbs demonstrates that the leg-length discrepancy has improved. Pelvic obliquity has almost improved (*dotted line*).

**Figure 3:**
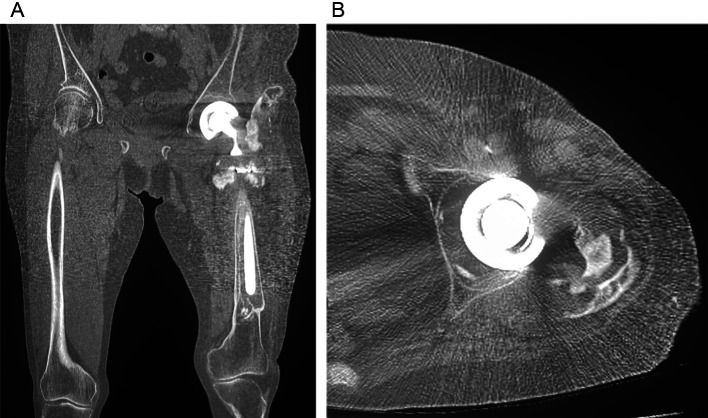
A computed tomography scan showing stem loosening with massive proximal femoral bone loss (A) and acetabular osteolysis (B).

An 85-year-old woman presented to our hospital with severe pain in her left hip joint. She weighed 47.1 kg, was 143 cm tall, and had a body mass index of 22.8 kg/m^2^. She had experienced a left-displaced femoral neck fracture approximately 30 years prior, and bipolar hemiarthroplasty (BHA) was performed in another hospital. Approximately 20 years after the first surgery, stem revision was performed in another hospital, the details of which are unclear. Ten years after revision surgery, the patient presented with severe left hip pain. She was unable to walk owing to pain and was transported in a wheelchair. A radiograph of the femur demonstrated stem loosening and massive bone loss from the proximal to the distal portion of the diaphyseal femur (Paprosky type IV) [5] with acetabular osteolysis (Figure 1A). A radiograph of the full-length lower limbs demonstrated a severe leg length discrepancy due to shortening of the femur (Figure 2A). Computed tomography revealed stem loosening with massive proximal femoral bone loss and acetabular osteolysis (Figures 3A and 3B). Microbial cultures of the joint were negative, so periprosthetic joint infections were excluded. Conversion THA after failed BHA with femoral reconstruction was performed using the following surgical techniques.

### Surgical procedure

**Figure 4:**
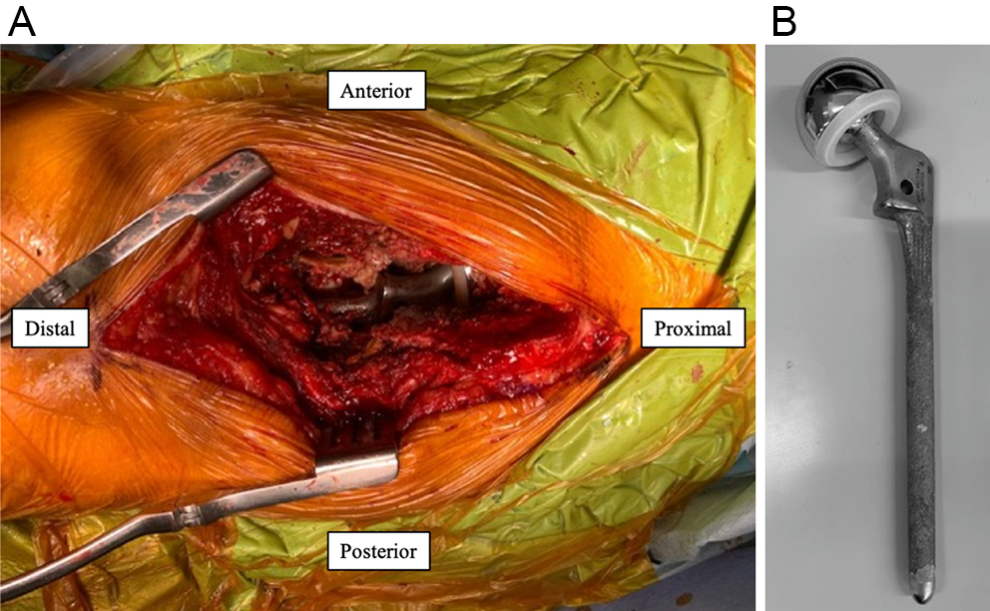
(A) The previous stem was easily removed due to looseness. Osseointegration is not observed around the stem. (B) Removed implant.

The patient was placed in the right lateral decubitus position during the procedure. Intraoperative fluoroscopy was performed. A direct lateral approach was used with a distal extension to the distal part of the previous stem. The atrophied gluteus medius and vastus lateralis muscles were exposed and split longitudinally along with their fibers. Subsequently, they were separated as anterior and posterior flaps to the femur. After dislocation, the previous stem was loose, it was easily removed after detaching from the surrounding scar tissue (Figures 4A and 4B). After the acetabulum was reamed, an optimally sized cementless cup (Trident, Stryker, Mahwah, NJ, USA) was placed in the appropriate position, aiming for inclination and anteversion angles of 40° and 20°, respectively.

**Figure 5:**
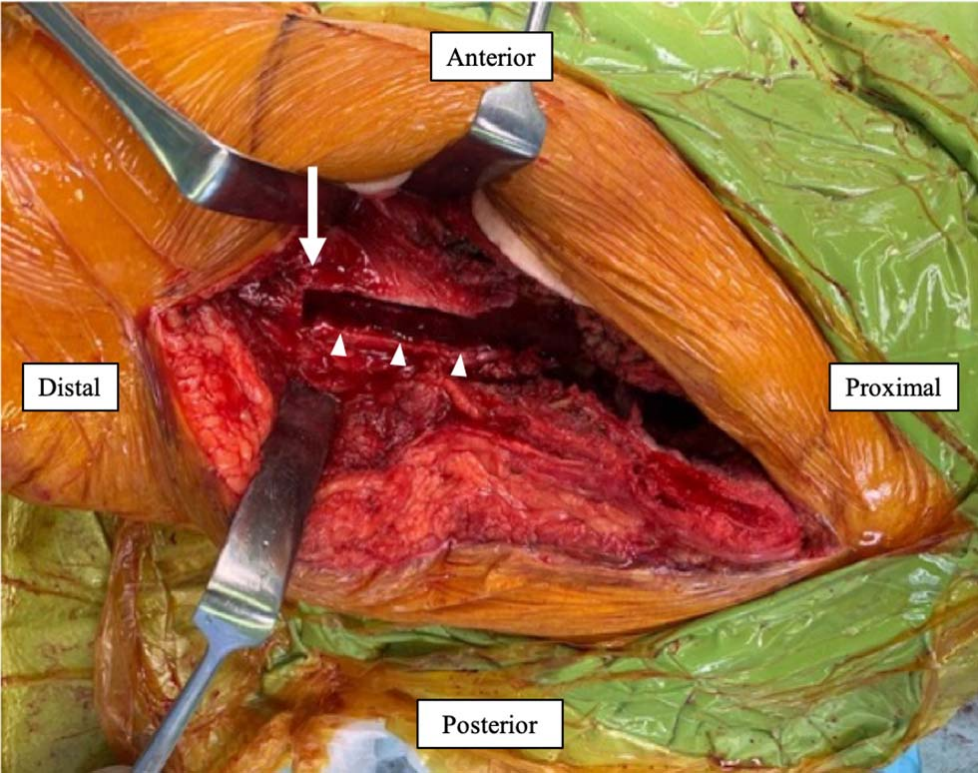
Osteotomy was performed such that the PFR stem was inserted as far into the intact part of the femur as possible (*white arrow*). After osteotomy, the remaining bone fragment of the proximal diaphyseal femur was longitudinally opened and separated without detaching it from the muscle (*white arrowheads*).

**Figure 6:**
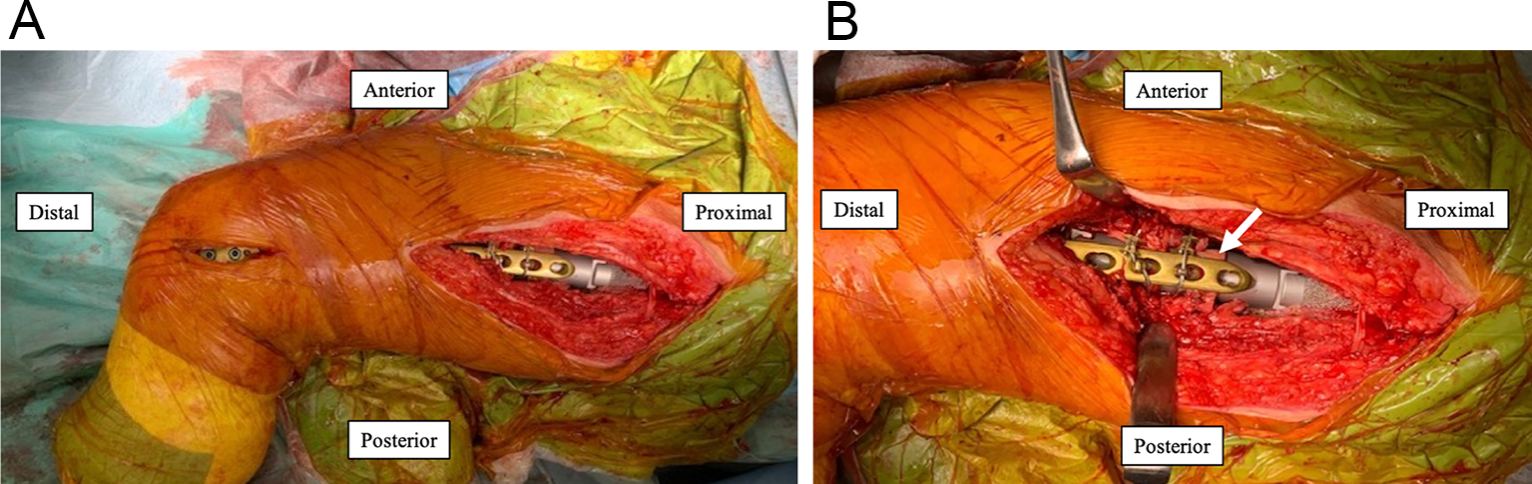
(A) A plate was inserted from the proximal incision to the distal incision along the periosteal surface of the bone using a minimally invasive plate osteosynthesis technique. A locking plate spanning from the PFR to the level of the femoral condyles was utilized. (B) A bone fragment extracted during prior osteotomy (*white arrow*) was placed between the PFR and locking plate such that they would not come into direct contact.

On the femoral side, osteotomy was performed so that the PFR stem would fit into the distal femoral host bone, with as few bone defects as possible (Figure 5). After osteotomy, the remaining bone fragment of the proximal diaphyseal femur was opened longitudinally and separated without detachment from the muscle (Figure 5). Several trial reductions were performed under direct and fluoroscopic observations to determine the appropriate length and version. When all of these parameters were satisfied, a PFR (GMRS Proximal Femoral, Stryker, Mahwah, NJ, USA) was inserted into the distal femoral host bone and fixed at the junction with cement. The remaining bone fragments of the proximal diaphyseal femur that had been opened longitudinally were used as onlay strut grafts to surround the prosthesis using a UHMWPE fiber cable (NESPLON Cable System, Alfresa Pharma Co., Osaka, Japan). In addition, a locking plate (LCP Distal Femur Plate, Johnson & Johnson, New Brunswick, New Jersey, USA) bridging the PFR and distal femoral host bone was added to reduce the torsional and compressive stresses on the bone cement prosthesis (Figure 6A). A free cortical bone fragment extracted during the prior osteotomy was placed between the PFR and locking plate to reduce the risk of generating metallic wear debris by preventing direct contact (Figure 6B). Dual-mobility articulation (MDM, Stryker, Mahwah, NJ, USA) was used to reduce the risk of dislocation. After implant placement, the anterior and posterior flaps of the gluteus medius and vastus lateralis were anatomically repaired and closed in a single layer. The flaps were reattached to the intertrochanteric region and PFR was performed as much as possible.

### Operative cautionary points

- Two experienced hip surgeons (HO and TB: >100 hip arthroplasties per year, respectively) performed this surgery. An experienced hip surgeon should participate in the surgery.
- A lateral approach was selected because all procedures, such as removal of the previous implant, THA conversion, PFR insertion, and plate placement, could be performed.
- To preserve hip joint stability, we attempted to avoid detaching as much soft tissue from the bone as possible.
- The osteotomy line was decided such that the PFR stem was inserted as far into the intact part of the femur as possible.
- In patients with massive bone loss and very poor bone quality, modular-type PFR with cementation should be the recommended option [6].
- Polymethylmethacrylate bone cement with tobramycin (1 g per 40 g bag of cement; Simplex with Tobramycin; Stryker) was used to prevent prosthetic joint infection and aseptic loosening [7].
- Dual-mobility articulation was used to reduce the risk of dislocation [4].
- A condylar-locking compression plate was selected to insert multiple bicortical screws distal to the PFR stem.
- A plate was inserted using a minimally invasive plate osteosynthesis technique to minimize soft-tissue injury and dissection and preserve blood supply to the bone fragments.
- A bone fragment was inserted between the metal implants to prevent them from touching directly.

### Indication for PFR with locking plate

The primary indication for this reconstruction method is poor femoral host bone quality or an insufficient amount of host bone in older adults. APCs are typically selected for young patients when adequate bone stock is needed for future revisions [8]. However, this reconstruction method may be utilized regardless of age instead of total femur replacement or resection arthroplasty in cases with an insufficient amount of host bone, such as after surgery for bone tumors.

### Postoperative course and follow-up

Full weight-bearing rehabilitation was started the day after surgery. Three weeks after surgery, the patient was able to walk with a cane. At the 5-year follow-up, she could walk steadily without complications; her modified Harris Hip Score had improved from 14.3 pre-surgery to 89.1 at the final follow-up. The postoperative range of motion in the left hip was 90° of flexion, 30° of internal rotation, 35° of external rotation, 45° of abduction, and 30° of adduction. A postoperative radiograph of the femur showed no signs of a radiolucent line, implant body problem, or bone resorption (Figure 1B). A postoperative radiograph of the full-length lower limbs demonstrated that the leg-length discrepancy had improved (Figure 2B).

## Discussion

Conversion THA after failed BHA with femoral reconstruction using PFR with a locking plate was performed for massive proximal femoral bone loss. This reconstructive technique may be useful for reducing high torsional and compressive stresses on bone cement prostheses, especially in cases of poor femoral host bone quality. Dual-mobility articulation is a suitable implant option for preventing postoperative dislocation.

PFR, APC, and long revision stems are the main treatment options for extensive poor proximal femoral bone stock quality [2]. For every reconstruction method, the postoperative functional results have been extremely satisfactory [2, 9, 10]. The complication rates for each reconstruction method are shown in Table 1. Although the use of long revision stems, including cementless and cement stems, is associated with an overall decrease in complication rates compared to other methods [10], when the quality of the bone is poor or the remaining bone is insufficient, such as in this case, revision stems cannot be used [2]. Furthermore, in these conditions, PFR is considered to increase the risk of loosening and PFF. Therefore, we devised a reconstruction method using PFR with a locking plate, rather than PFR alone. As there are very few effective reconstruction options for these conditions, this reconstruction method or APC was considered.

**Table 1:** Complication rates for different femoral reconstruction methods for massive proximal femoral bone loss.

Reconstruction method	Reoperation	Revision	Dislocation	Infection	Aseptic loosening	Periprosthetic fracture
Proximal femoral replacement [2]	20.3%	15.4%	10.2%	7.4%	5.0%	2.4%
Allograft prosthetic composite [9]	59.9%	26.5%	11.1%	6.9%	5.6%	Allograft fracture: 5.6%
						Supracondylar femoral fracture: 2.8%
Cementless revision stem [10]	11.4%	4.8%	2.0%	0.6%	0.7%	0.2%
Cemented long revision stem [10]	–	2.9%	0.1%	0.3%	1.7%	0.6%

In this case, because the patient was an older adult, it was important to acquire walking ability early after surgery to maintain the activities of daily living. However, because of the poor femoral host bone quality, robust femoral reconstruction was necessary. Therefore, PFR with a locking plate, which leads to more robust fixation without postoperative weight-bearing restrictions, was considered more suitable than APC, which requires such restrictions. APC has the advantage of restoring bone stock but is technically demanding and has inherent complications, which include periprosthetic bone resorption, non-union at the graft-host bone junction, fractures, infection, and disease transmission risk [1]. On the other hand, PFR, a type of megaprosthesis implantation, is a simple but non-biologic surgical procedure with limited longevity [1]. One of the issues with megaprostheses in terms of longevity is aseptic loosening of the femoral components [3]. Because of the diaphyseal cement fixation, the junction of the bone cement prosthesis is subjected to high torsional and compressive stresses, resulting in early loosening [3]. Hence, in this case, we attempted to reduce these stresses by adding a locking plate bridging the PFR and distal femoral host bone. However, further follow-up is required to assess its effectiveness.

No studies have conducted a biomechanical assessment of this reconstruction method. Therefore, we referred to the biomechanical study of PFF. Walcher et al. established a safe distance from the distal femur locking plate to the tip of a stable femoral stem [11]. To avoid stress risers, an overlap of at least 6 cm is recommended in osteoporotic bone [11]. In this case, a plate was placed on the femur such that it overlapped the PFR by at least 6 cm in the proximal direction from the junction of the bone cement prosthesis where stress was the highest. Another study investigated three different combinations of fixation (proximal unicortical locking screws alone, proximal cables and unicortical locking screws, and proximal cable alone) with distal bicortical-locking screws for simulated Vancouver type B1 [12]. PFFs around a stable stem alter the stiffness and strain of fixation [13]. Regarding stiffness, proximal unicortical locking screws alone provided the stiffest form [13]. Regarding strain, it was considerably lower on the plate and femur with proximal cables alone than other methods [13]. In this case, a locking plate was fixed with wires proximal to the bone-cement-prosthesis junction and with bicortical-locking screws distal to the bone-cement-prosthesis junction. Hence, in this case using combined fixation using proximal cables and distal screws, the stiffness of this construct may decrease, but the strain on the PFR, remaining femoral bone, and locking plate may be reduced. The locking plate was fixed to assist in the fixation of the PFR and remaining femoral bone; this strategy may be beneficial for long-term results. Despite not using a biomechanical approach, a previous study reported that internal fixation using a plate extending the length of the femur to the height of the femoral condyles for PFFs around a well-fixed arthroplasty stem reduces the incidence of non-union and refracture [14]. They also reported that because the short plate serves as a stress riser in osteoporotic bone, some patients had experienced refracture distal to the previous fixation construct requiring reoperation. [14]. A laterally based plate spanning the length of the femur to the level of the femoral condyles not only increases biomechanical stability but also reduces the risk of refracture distal to previous fixation [14]. Therefore, in this case, using a long locking plate that spans from the PFR to the level of the femoral condyles may have potentially reduced the risk of PFF. However, a biomechanical study is required to examine the mechanism of load transmission via the femur reconstructed using this method.

## Conclusion

Femoral reconstruction using PFR with a locking plate is particularly beneficial for older patients with massive proximal femoral bone loss. Although this reconstruction method may reduce complications, particularly on the femoral side, including stem loosening and PFFs, careful monitoring through long-term follow-up is essential. Further validation of this reconstruction method through biomechanical studies is also required. In future, it will be important to increase the number of reconstruction cases using this technique and accumulate data on its indications and long-term results. We plan to use this data to improve outcomes of patients suffering from massive proximal femoral bone loss.

## Data Availability

Data is available upon request from the corresponding author.
